# International Guidelines on Conscious Sedation in Pediatric Dentistry: A Comparative Analysis and Evidence Mapping Study

**DOI:** 10.3390/jcm15072673

**Published:** 2026-04-01

**Authors:** Carolina Marques, Mafalda Dinis, João Botelho, Vanessa Machado, Luísa Bandeira Lopes

**Affiliations:** 1 Egas Moniz School of Health & Science, Campus Universitário, Quinta da Granja, Caparica, 2829-511 Almada, Portugal; 2 Egas Moniz Center for Interdisciplinar Research (CiiEM); Egas Moniz School of Health & Science, Campus Universitário, Quinta da Granja, Caparica, 2829-511 Almada, Portugalllopes@egasmoniz.edu.pt (L.B.L.)

**Keywords:** pediatric dentistry, conscious sedation, clinical practice guidelines, behavior management, nitrous oxide

## Abstract

Conscious sedation is widely used in pediatric dentistry to manage dental anxiety, behavioral difficulties, and systemic diseases that compromise patient compliance with dental care. Despite its clinical importance, international recommendations vary considerably. Objective: To conduct a comparative analysis and evidence mapping of international clinical practice guidelines on conscious sedation in pediatric dentistry. Methods: A comparative guideline analysis and evidence mapping study was performed. Electronic searches were conducted in PubMed (MEDLINE), Scopus, EMBASE, Cochrane Database of Systematic Reviews, Web of Science, LILACS, SciELO, TRIP, and OpenGrey up to December 2023. Guidelines issued by recognized professional or governmental organization addressing conscious sedation in pediatric dentistry were included. Predefined domains were analyzed, including indications, contraindications, pharmacological agents, dosages, routes of administration, monitoring, discharge criteria, and professional training. Data were synthesized descriptively and graphically mapped to illustrate coverage patterns. Results: Twelve international guidelines were included. Complete convergence (100%) was observed in core safety domains, such as patient assessment, monitoring, and professional training. A high agreement was found for discharge criteria (91.67%) and contraindications (83.33%). However, substantial variability emerged in pharmacological protocols, with only 16.67% of guidelines providing comprehensive drug and dosage descriptions. Routes of administration and emergency equipment recommendations were inconsistently reported, appearing in 66.67% and 50% of guidelines, respectively. Conclusions: Although foundational safety principles are consistently addressed, significant heterogeneity persists in pharmacological and procedural recommendations. This variability may contribute to differences in practice and uncertainty among practitioners. Greater international harmonization of guidelines may improve consistency, enhance clinical decision-making, and strengthen patient safety in pediatric dental care. Clinical Relevance: Identifying areas of convergence and variability across international guidelines may support the development of more standardized sedation protocols and promote safer evidence-based clinical practice in pediatric dentistry.

## 1. Introduction

Dental fear and anxiety are highly prevalent among children and adolescents and represent significant barriers to the delivery of safe and effective dental care [1]. These behavioral challenges may compromise treatment quality, increase procedural risk, and negatively affect long-term oral health outcomes. Conscious sedation is a minimal drug-induced depression of consciousness during which patients respond to verbal commands or light tactile stimulation while maintaining protective reflexes and adequate spontaneous ventilation [2]. In addition, the patient can independently maintain an open mouth and retain adequate function of protective reflexes [1]. Conscious sedation has therefore become an essential ally for behavior control in pediatric dentistry, particularly for anxious and non-cooperative patients, and systemic diseases that affect patient compliance [1,3].

Conscious sedation is indicated in children with low coping ability, such as fear and anxiety regarding dental treatment, behavioral management problems and in children who, due to physical or mental limitations, are unable to cooperate [3]. However, pediatric sedation is not without risk. Adverse events, such as respiratory depression, airway obstruction, hypoventilation, and cardiopulmonary complications, highlight the need for strict safety protocols and adequate professional training [2].

To address these risks, multiple professional and governmental organizations worldwide have developed clinical practice guidelines on conscious sedation in pediatric dentistry [2,3]. Numerous agents can be used, including benzodiazepines, nitrous oxide, and other drugs. These can be administered by a variety of routes, such as orally, intravenously, intranasally, and in variety of combinations and doses [1].

However, differences in pharmacological recommendations, dosing protocols, monitoring and safety measures may contribute to variability in clinical practice across regions.

Variability across international guidelines may lead to inconsistencies in clinical decision-making, differences in training standards, and potential implications for patient safety. In pediatric dentistry, where sedation carries inherent risks, the lack of standardized recommendations may contribute to uncertainty among practitioners and variability in clinical outcomes [4,5]. Clinical guidelines play a key role in supporting decision-making and improving healthcare quality; however, inconsistencies may negatively influence clinical practice and patient outcomes [6,7].

The increasing globalization of dental education and professional mobility has highlighted the importance of harmonizing clinical recommendations across regions. Differences in national guidelines may influence training standards, clinical decision-making, and safety protocols adopted in pediatric dental practice. Therefore, understanding how these guidelines converge or diverge is essential for identifying areas where international consensus exists and where further standardization may be required. Comparative analyses of clinical guidelines can provide valuable insights into the consistency of recommendations and help clinicians identify best practices supported by broader international literature.

Despite the existence of numerous international guidelines, structured comparative analyses of their scope, consistency, and content coverage remain limited. To address this gap, this study evaluated predefined clinical domains commonly included in sedation guidelines, such as indications, contraindications, pharmacological protocols, monitoring, and discharge criteria. These domains represent key components of safe and effective sedation practice. Therefore, this study aimed to conduct a comparative guideline analysis and evidence mapping of international clinical practice guidelines on conscious sedation in pediatric dentistry, identifying areas of convergence, variability, and potential gaps in recommendations.

## 2. Materials and Methods

This study was designed as a descriptive comparative guideline analysis and evidence-based mapping study. This methodological approach has been used previously to systematically compare the scope, consistency, and content of clinical practice guidelines across different healthcare fields [4,5].

This study focused on conscious sedation in pediatric dentistry, considering international clinical practice guidelines as the primary source of analysis. The outcomes of interest included the scope, consistency, and variability of recommendations regarding indications, contraindications, pharmacological agents, dosages, routes of administration, monitoring, emergency, discharge criteria, and professional training.

The study aimed to address the following research questions:

(1) What areas of convergence and divergence exist among international clinical practice guidelines on conscious sedation in Pediatric Dentistry?
(2) Which clinical domains demonstrate consistency, variability, or gaps in pharmacological and safety recommendations?

### 2.1. Eligibility Criteria

The inclusion criteria were as follows: (1) guidelines issued by a recognized entity; (2) addressing conscious sedation in pediatric dentistry. The exclusion criteria were as follows: (1) guidelines exclusively focused on general anesthesia; (2) documents not issued by recognized professional or governmental entities; (3) opinion papers, narrative reviews, or educational materials without a formal guideline structure; and (4) guidelines not applicable to dental settings. No restrictions were applied regarding the year of publication or language.

### 2.2. Information Sources and Search Strategy

Electronic data search was performed in the following electronic databases: PubMed (via Medline), Scopus, Cochrane Database of Systematic Reviews, Scielo (Scientific Electronic Library Online), EMBASE (The Excerpta Medica Database), LILACS (Latin-American scientific literature in health sciences), TRIP (Turning Research Into Practice), Web of Science, and OpenGrey. The final search was performed in December 2023.

The search strategy was designed to ensure broad coverage of international recommendations related to conscious sedation in pediatric dental settings. Both controlled vocabulary terms (e.g., MeSH) and free-text keywords were used to maximize sensitivity, and the search strategy was adapted to the indexing systems of each database. The reference lists of relevant documents were screened to identify additional guidelines that might not have been retrieved through database searches. When multiple versions of the same guideline were identified, the most recent version was included in the analysis. The selection process aimed to capture documents developed by recognized professional organizations or governmental bodies that provide recommendations applicable to clinical dental practice. The following search syntax was applied: (“conscious sedation”[MeSH] OR sedation OR “conscious sedation”) AND (pediatric[MeSH] OR paediatric OR pediatric) AND guidelines.

### 2.3. Study Selection

Two researchers (MD and CM) independently performed the identification, screening, and selection of clinical practice guidelines on conscious sedation in pediatric dentistry, developed by recognized entities across different continents (Africa, Asia, the Americas, Europe, and Oceania). They then independently analyzed the titles and abstracts. Any guideline considered potentially eligible by either researcher was ordered as a full-text and independently screened by the researchers. All disagreements were resolved through discussion with a third researcher (LBL).

### 2.4. Data Extraction Process and Data Items

Two researchers (MD and CM) independently extracted predefined data domains, including objectives, definitions of sedation levels, indications, contraindications, adverse effects, pharmacological agents and dosages, routes of administration, professional training requirements, facilities and equipment, patient assessment and preparation, documentation, fasting, monitoring, emergency equipment, and discharge criteria. All disagreements were resolved through discussion with a third researcher (LBL).

A descriptive quantitative synthesis was performed to calculate the proportion of guidelines addressing each predefined domain. Guidelines were grouped by continent and chronologically categorized. Data are presented in a tabular format and graphically mapped to visualize coverage patterns, convergence, divergence, and temporal distribution.

The methodological quality of individual guidelines was not formally appraised (e.g., using the AGREE II instrument), because the primary objective of this study was to map the presence and coverage of clinical domains rather than to evaluate the methodological rigor of guideline development. This approach allows the identification of thematic convergence and variability across international recommendations. This methodology was designed to ensure transparency and reproducibility in the identification and comparison of international clinical practice guidelines.

## 3. Results

Twelve clinical practice guidelines issued by recognized professional or governmental entities were included in the analysis [3,8,9,10,11,12,13,14,15,16,17,18]. The guidelines on conscious sedation in pediatric dentistry were developed by recognized entities from different continents (Africa, Asia, Americas, Europe, and Oceania). The characteristics are summarized in Table 1. These guidelines were included to enable a comparison of their recommendations across predefined clinical domains. Representing the African continent, we have the South African guideline by the South African Society of Anaesthesiologists (SASA) of 2016 [8]. Representing the Americas, we have Canada (North America) with a guideline from the Royal College of Dental Surgeons of Ontario in 2018 [9], Chile (South America), with a guideline developed by the Department of Oral Health in 2021 [10], and the United States of America (North America) with a guideline from the American Academy of Pediatric Dentistry in 2019 [11]. From the Asian continent, the selected guidelines include the Chinese guideline, developed by the Hong Kong Society of Paediatric Dentistry in 2009 [12]; the Indian guideline from the Indian Health Services in 2007 [13]; the Japanese guideline, prepared by The Japanese Dental Society of Anesthesiology in 2017 [14]; and finally, the guideline from Singapore, elaborated by the Academy of Medicine, Singapore, in 2021 [15]. Europe presents a guideline from The European Academy of Paediatric Dentistry in 2021 [3], but it is also represented by Scotland with a guideline from The Scottish Dental Clinical Effectiveness Programme in 2017 [16] and the UK with a guideline from the Royal College of Surgeons in 2002 [17]. Oceania is represented by Australia, with a guideline developed by the Australian and New Zealand College of Anaesthetists & the Faculty of Pain Medicine in 2022 [18]. These topics include the aims of sedation, definition of sedation levels, indications, contraindications, adverse effects, the approach to drugs such as nitrous oxide, midazolam, diazepam, and chloral hydrate, their dosages, routes of administration, information on staff education and training, necessary facilities and equipment, patient assessment and preparation, required documents and records, fasting requirements, monitoring, emergency equipment, and discharge criteria.

**Figure 1:**
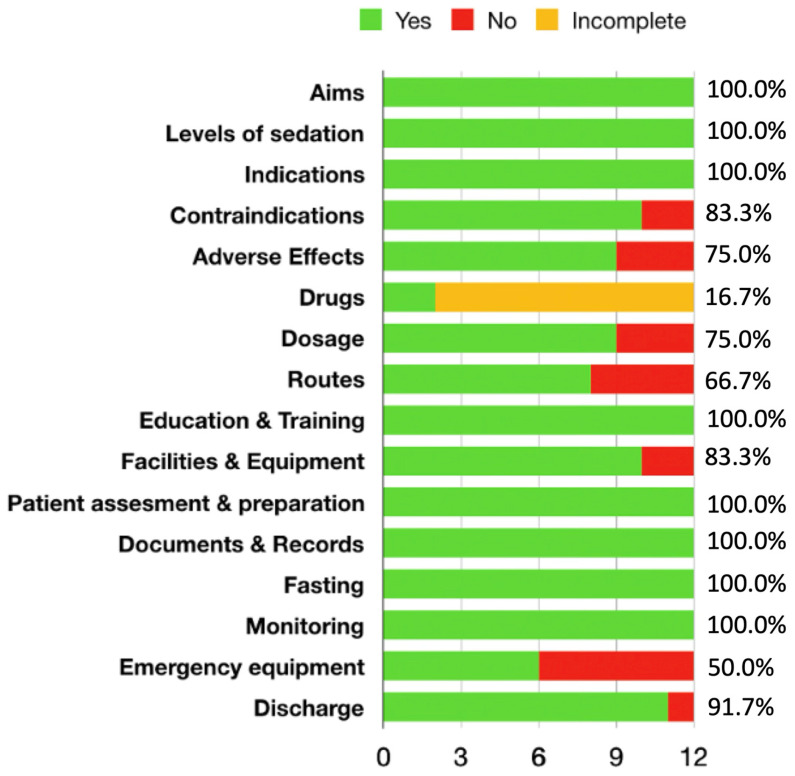
Distribution of topic coverage across the included clinical practice guidelines.

As shown in Figure 1, several domains were addressed in 100% of the included guidelines, namely objectives of sedation, definitions of sedation levels, indications, staff education and training, patient assessment and preparation, documentation, fasting requirements, and monitoring. Figure 1 also illustrates variability across individual guidelines in the coverage of specific domains, particularly in pharmacological and emergency-related aspects.

The guidelines were compared in terms of their approaches to several predefined domains, including the aims of sedation, definitions of sedation levels, indications, contraindications, adverse effects, pharmacological agents and dosages, routes of administration, staff education and training, facilities and equipment, patient assessment and preparation, documentation and records, fasting requirements, monitoring, emergency equipment, and discharge criteria (Table 1).

**Figure 2:**
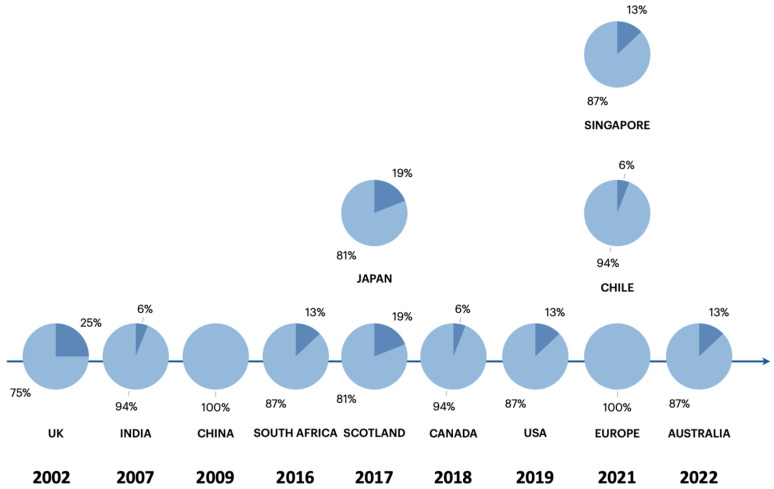
Temporal evolution of guidelines and the number of topics addressed in each one.

The distribution of topic coverage across the included guidelines is illustrated in Figure 1, and the temporal distribution and overall domain coverage are shown in Figure 2.

### 3.1. Domain Coverage and Convergence

Complete convergence (100%) was observed in the following domains: objectives of sedation, definitions of sedation levels, indications, staff education and training, patient assessment and preparation, documentation and records, fasting requirements, and monitoring (Figure 1). High levels of coverage were identified for discharge criteria (91.67%), contraindications (83.33%), and facilities and equipment (83.33%). Adverse effects and drug dosages were addressed in 75% of the guidelines. Routes of administration were described in 66.67% of the documents, while emergency equipment was explicitly mentioned in 50% of the included guidelines.

Although all guidelines referred to pharmacological agents used in conscious sedation, only 16.67% provided comprehensive descriptions of nitrous oxide, midazolam, diazepam, and chloral hydrate, together with dosage specifications (Table 1). These findings highlight that, while core safety domains are consistently addressed across guidelines, substantial variability persists in pharmacological and procedural aspects. In particular, the limited consistency in drug selection, dosage recommendations, and routes of administration reflects a lack of standardized protocols, which may contribute to heterogeneity in clinical practice.

### 3.2. Overall Completeness of Guidelines

The overall coverage of the predefined domains ranged from 75% to 100% across the included guidelines (Figure 2). Two guidelines addressed all the analyzed domains (100% coverage): the American [11] and the European guidelines [3].

Figure 2 illustrates the variability in content breadth and temporal distribution of publications. More recent guidelines have generally demonstrated broader coverage of safety and procedural domains; however pharmacological details remain inconsistent across documents.

## 4. Discussion

This comparative analysis demonstrates that, although international guidelines on conscious sedation in pediatric dentistry share fundamental safety principles, considerable heterogeneity persists in pharmacological recommendations and procedural details. These findings are consistent with previous studies that have reported variability across clinical practice guidelines in different healthcare fields, highlighting the challenges in achieving standardization and harmonization of recommendations.

Two guidelines are frequently regarded as reference frameworks: the American guidelines in “Guidelines for Monitoring and Management of Pediatric Patients Before, During, and After Sedation for Diagnostic and Therapeutic Procedures” [11], and the European “Best Clinical Practice Guidance for Conscious Sedation of Children Undergoing Dental Treatment” [3]. Both address core domains, including sedation objectives, definitions of sedation levels, adverse effects, staff training, facilities and equipment, patient assessment, documentation, fasting, monitoring, emergency preparedness, and discharge criteria. However, the American guidelines do not explicitly detail contraindications or routes of administration and provide limited pharmacological specificity regarding benzodiazepines [11], whereas the European guidelines offer more structured drug and dosage descriptions [3].

Overall, convergence was observed in approximately half of the predefined domains, particularly those related to safety governance and patient assessment. In contrast, divergence was most evident in drug selection, dosage range, and preferred route of administration.

### 4.1. Sedation Goals and Definitions

Consensus is achieved in the definition of moderate sedation, also known as conscious sedation. It is defined as a drug-induced depression of consciousness during which patients respond purposefully to verbal commands or after light tactile stimulation. No interventions are required to maintain patent airway, and spontaneous ventilation is adequate. Cardiovascular function is usually maintained. The European guideline does not describe the other levels of sedation, and three others are in line with this one [13,14,17], but the others are based on the American one and converge in their definitions [8,9,10,11,12,13,15,18]. Minimal sedation is a drug-induced state during which patients respond normally to verbal commands. Although cognitive function and coordination may be impaired, ventilatory and cardiovascular functions are unaffected. Deep sedation is described as a drug-induced depression of consciousness during which patients cannot be easily aroused but respond purposefully after repeated verbal or painful stimulation (e.g., purposefully pushing away noxious stimuli). Reflex withdrawal from a painful stimulus is not considered a purposeful response and is more consistent with a state of general anesthesia.

Therefore, all guidelines converge in defining the primary goals of sedation as relief of anxiety and fear, minimization of discomfort, and facilitation of safe treatment completion [3,8,9,10,11,12,13,14,16,17,18]. Most guidelines adopt the American classification of minimal, moderate, and deep sedation [8,9,10,11,12,13,15,18]. The European document [3] focuses primarily on conscious sedation without extensively detailing other levels, while a limited number of guidelines follow a similar approach [13,14,17]. Despite minor structural differences, conceptual consistency remains strong.

### 4.2. Indications and Patient Selection

There is broad agreement that American Society of Anesthesiologists (ASA) physical status classification I and II patients are appropriate candidates for conscious sedation [3,8,9,10,11,12,15,16,17,18]. However, variability exists regarding minimum age thresholds, which range from 1 to 5 years depending on the guideline [3,8,9,12,15,18].

It is important to note that the Japanese guidelines are the only ones to assume that most patients are strong candidates for sedation; however special attention must be provided to patients who require special care. It is important to note that this guideline only addresses intravenous sedation [14].

Additional caution is warranted in patients with ASA III–IV and children with special healthcare needs, airway abnormalities, or sleep disorders [3,8,9,10,11,12,13,15,16,17,18]. Nitrous oxide-specific indications are detailed in only two guidelines [3,12], reflecting limited uniformity in drug-specific patient selection criteria.

### 4.3. Contraindications and Adverse Effects

Absolute contraindications are rarely defined. Instead, guidelines recommend individualized risk assessment and consultation with medical specialists when significant comorbidities are present. The European guidelines provide a structured list of contraindications [3], whereas the American guidelines do not explicitly address this domain [11]. Commonly reported adverse effects include respiratory depression, airway obstruction, apnea, nausea, vomiting, and oversedation [3,10,11,12,13,14,15,16,17]. Less frequently reported events include laryngospasm, cardiovascular instability, and loss of protective reflexes. Despite differences in reporting frequency, there is general agreement regarding potential respiratory risks.

### 4.4. Pharmacological Variability

Pharmacological recommendations represent the most heterogeneous domain. Nitrous oxide is referenced in nearly all guidelines, except the Japanese document, which focuses exclusively on intravenous sedation [14]. While several guidelines consider inhalation sedation a preferred modality [8,10,11,13,16], there is no consensus regarding optimal concentration thresholds. Some recommend maximum concentrations of 50% [8,12,13], whereas others accept concentrations up to 70% [3,8,11,15]. Oral midazolam is widely described, yet recommended dosages vary between 0.25 and 0.5 mg/kg, with maximum doses ranging from 7.5 mg to 15 mg [3,8,9]. Intravenous dosing protocols also differ substantially [3,8,9,14]. Similarly, diazepam and chloral hydrate demonstrate inconsistent indications and dosage ranges across guidelines [3,8,11,17]. These discrepancies prevent the identification of a universally endorsed pharmacological protocol.

### 4.5. Education, Facilities, and Monitoring

Strong convergence exists in training requirements. All guidelines emphasize the need for formal theoretical and practical education, as well as competence in airway management and the ability to rescue patients from sedation levels deeper than intended [3,8,9,10,11,12,13,14,15,16,17,18]. Facility and equipment requirements largely align with the American framework (11), including monitoring equipment, emergency preparedness systems, and oxygen delivery capability [3,8,9,10,11,12,13,15,16,17,18]. Monitoring parameters—pulse oximetry, blood pressure, heart rate, and respiratory rate—are consistently recommended [3,8,9,10,11,12,13,14,15,16,17,18].

### 4.6. Fasting and Discharge Criteria

Fasting recommendations generally align with the American guideline [7], advocating 2 h for clear fluids and 6 h for solids [8,9,10,11,12,14,15,17,18]. However, European guidelines suggest that nitrous oxide sedation may not require fasting in low-risk cases [3], illustrating procedural divergence. Discharge criteria demonstrate high consistency across guidelines [3,8,9,10,11,12,13,14,15,16,17,18], emphasizing stable vital signs, airway patency, adequate hydration, return of consciousness, and supervision by a responsible adult.

In summary, the findings of this comparative analysis highlight that while international guidelines share strong agreement in fundamental safety principles, important inconsistencies remain in pharmacological recommendations and procedural details. These variations may contribute to differences in clinical practice across regions and potentially influence patient safety and treatment standardization. Greater international alignment of evidence-based recommendations could support more consistent sedation practices and improve the safety of pediatric dental care worldwide. From a clinical perspective, variability in sedation protocols may create uncertainty among practitioners, particularly in settings where access to specialized training or institutional protocols may be limited. Differences in recommended pharmacological agents, dosage ranges, and routes of administration may also influence practitioner confidence and clinical decision-making [3,8,9,10,11,12,13,14,15,16,17,18]. In pediatric dentistry, where patient safety is a critical priority, the presence of clear and consistent guidance is essential to support safe sedation practices [2,3,11]. Furthermore, international comparisons of guidelines may contribute to the identification of core domains that should be consistently addressed in future guideline development. Elements such as patient assessment, monitoring standards, professional training requirements, and discharge criteria demonstrated strong convergence across documents, suggesting that these domains represent widely accepted safety foundations [3,8,9,10,11,12,13,14,15,16,17,18]. In contrast, pharmacological recommendations and dosage specifications showed greater variability, highlighting areas where additional evidence synthesis and international collaboration may be beneficial [3,8,9,11,12,14,17,18]. Future guideline development efforts may benefit from greater international collaboration among professional societies, with the aim of improving consistency of recommendations while still allowing adaptation to regional healthcare systems and regulatory frameworks [3,11,18].

The comparative and evidence mapping approach used in this study provides a structured overview of the current landscape of clinical practice guidelines, allowing the identification of areas of convergence and divergence. This methodology contributes to a better understanding of how recommendations are distributed across domains and highlights priorities for future guideline development and harmonization.

### 4.7. Limitations

This study had some limitations. First, the lack of a formal risk of bias assessment (e.g., using the AGREE II instrument) represents a limitation, as it may influence the interpretation of guideline quality and validity of comparisons.

Second, despite a comprehensive search strategy without language or date restrictions, some relevant national or non-indexed guidelines may not have been identified, and updates published after 31 December 2023 were not considered. Third, the analysis focused on the presence or absence of predefined domains and did not evaluate the strength of evidence supporting individual recommendations. Finally, heterogeneity in scope across guidelines—ranging from dentistry-specific pediatric documents to broader procedural sedation frameworks—may have influenced differences in content coverage.

## 5. Conclusions

This comparative guideline analysis and evidence mapping study demonstrates that international clinical practice guidelines on conscious sedation in Pediatric Dentistry show strong convergence in core safety domains, including patient assessment, monitoring, professional training, and discharge criteria. However, substantial heterogeneity persists in pharmacological recommendations, particularly regarding drug selection, dosage ranges, and routes of administration. From a clinical perspective, this variability may lead to uncertainty among practitioners and inconsistencies in sedation practices, particularly in settings lacking standardized institutional protocols. Clinicians should therefore prioritize guidelines with more comprehensive safety frameworks and exercise caution when applying pharmacological recommendations that lack consistency across documents. The findings of this study highlight the need for greater international harmonization of sedation guidelines, particularly in pharmacological protocols, to improve consistency in clinical decision-making and enhance patient safety.

Future research should focus on developing standardized, evidence-based pharmacological recommendations and evaluating the clinical outcomes associated with different sedation protocols. Additionally, greater international collaboration between professional societies may support the development of more unified and adaptable guidelines for pediatric dental sedation.

## Figures and Tables

**Table 1:** Comparison table of information contained in international guidelines on conscious sedation in Pediatric Dentistry, issued by recognized entities. (✓ (green) indicates the presence of the item in the guideline; ✕ (red) indicates its absence).

		Africa	American		Asia					Europe			Oceania
		South African	Canada	Chile	USA	China	India	Japan	Singapore	EU	Scottish	UK	Australia
Aims of sedation		✓	✓	✓	✓	✓	✓	✓	✓	✓	✓	✓	✓
Definition levels of sedation		✓	✓	✓	✓	✓	✓	✓	✓	✓	✓	✓	✓
Indications		✓	✓	✓	✓	✕	✓	✓	✓	✓	✓	✓	✓
Contraindications		✓	✓	✓	✕	✕	✓	✓	✓	✓	✓	✓	✕
Adverse effects		✓	✕	✓	✓	✕	✓	✓	✕	✓	✕	✓	✓
Drugs	Nitrous Oxide	✓	✓	✓	✓	✓	✓	✕	✓	✓	✓	✓	✓
	Midazolam	✓	✓	✕	✕	✕	✕	✓	✕	✓	✓	✓	✕
	Diazepam	✕	✓	✕	✕	✕	✕	✓	✕	✓	✕	✓	✕
	Chloral hydrate	✓	✕	✕	✓	✕	✕	✕	✕	✓	✕	✓	✕
Dosage		✓	✓	✕	✓	✕	✓	✓	✕	✓	✕	✕	✕
Routes		✓	✓	✓	✕	✓	✓	✕	✕	✓	✓	✓	✕
Education and Training		✓	✓	✓	✓	✓	✓	✓	✓	✓	✓	✓	✓
Facilities and equipment		✓	✓	✓	✓	✓	✓	✕	✓	✓	✓	✕	✓
Patient assessment and preparation		✓	✓	✓	✓	✓	✓	✓	✓	✓	✓	✓	✓
Documents and records		✓	✓	✓	✓	✓	✓	✓	✓	✓	✓	✓	✓
Fasting		✓	✓	✓	✓	✓	✕	✓	✓	✓	✓	✓	✓
Monitoring		✓	✓	✓	✓	✓	✓	✓	✓	✓	✕	✓	✓
Emergency equipment		✕	✓	✕	✓	✓	✕	✓	✕	✓	✕	✓	✓
Discharge		✓	✓	✓	✓	✓	✓	✓	✓	✓	✓	✕	✓

## Data Availability

No new data were created or analyzed in this study.
